# Efficacy and safety of traditional Chinese medicine decoction combined with chemotherapy in the treatment of advanced colorectal cancer: A protocol of a systematic review and meta-analysis

**DOI:** 10.1097/MD.0000000000023952

**Published:** 2021-01-22

**Authors:** Shufan Liao, Xue-li Jia, Yan Yang, Yu-xiang Sun, Si-miao Gong, Min Li

**Affiliations:** a School of Health Preservation and Rehabilitation, Chengdu University of Traditional Chinese Medicine; b School of Acupuncture-Moxibustion and Tuina, The Third Affiliated Hospital, Chengdu University of Traditional Chinese Medicine.

**Keywords:** colorectal cancer, meta-analysis, protocol, systematic review, traditional Chinese medicine decoction

## Abstract

**Background:**

Colorectal cancer has become a major chronic and difficult disease endangering human health. After thousands of years of precipitation, traditional Chinese medicine in China is now also being applied in clinical treatment, with its unique advantages in the treatment of cancer. However, the efficacy of traditional Chinese medicine in the treatment of advanced colorectal cancer still cannot reach consensus in the world. Therefore, the aim of this study was to provide a scheme to evaluate the efficacy and safety of traditional Chinese medicine decoction in the treatment of advanced colorectal cancer, thus providing clinical decision-making.

**Methods and analysis:**

The systematic review and meta-analysis will be conducted in accordance with the Preferred Reporting Items for Systematic Review and Meta-Analysis (PRISMA) guidelines. The following 8databases will be searched: China National Knowledge Infrastructure (CNKI), China Biology Medicine (CBM), Wan Fang Data, the Chinese Science and Technology Periodical Database (VIP), PubMed, Cochrane Library, Embase, and Web of Science. Relevant data will be performed by Revman 5.3 software provided (Cochrane Collaboration) and Stata 14.0 statistical software.

**Results:**

The results of this systematic review and meta-analysis will be published in a peer-reviewed journal.

**INPLASY registration number:**

INPLASY202080102.

## Abstract 2

Supplemental Digital Content is available in the text

## 1 Introduction

Although the mortality of colorectal cancer is decreasing, it is still a digestive tract malignant tumor which seriously threatens human health.^[1]^ In 2018, it had more than 1,800,000new cases worldwide, with an estimated death rate of 880,000 people, with a third incidence rate and second mortality rate.^[2]^According to the estimated numbers of new invasive cancer cases in the United States in 2020,Colorectal cancer is one of the most frequently diagnosed tumors in both sexes.^[1,3]^ The early symptoms of colorectal cancer is more mild or not obvious,^[4]^ so it is not easy to be detected by patients, resulting in delayed treatment. For such patients, prolonging survival time^[5]^ and alleviating pain^[6]^ are the treatment goals. Chemotherapy plays an important role^[7]^ in the treatment of advanced colorectal cancer. The commonly used chemotherapy regimens include m FOLFOX6, FOLFIRI, Cape OX and capecitabine.^[8–10]^

Traditional Chinese medicine, as a treasure of China, believes that the treatment of advanced colorectal cancer should be strengthening the body and attacking pathogenic factors,^[11,12]^ which is consistent with prolonging the survival period and alleviating the pain of patients. Through long-term treatment, traditional Chinese medicine can achieve the effect of overall regulation. At present, studies have shown the anticancer mechanism of the commonly used Chinese herbal decoction for colorectal cancer.^[13,14]^ Jianpi Xiaoai recipe can up regulate E-cadherin and down regulate vimentin table by mediating TGF–β.^[15]^ Jianpi Jiedu recipe inhibits the expression of snail / E-cadherin in colorectal cancer induced by transforming growth factor (TGF -) and EMT-β/ Smad.^[16]^

Although some researchers seem to have achieved a certain effect in the treatment of advanced colorectal cancer with traditional Chinese medicine decoction combined with chemotherapy, the research design is not strictly in accordance with the randomized controlled trials, the evaluation standards of curative effect are inconsistent, and the research conclusions obtained are not repeatable and reliable. In order to evaluate the efficacy of traditional Chinese medicine decoction in the treatment of advanced colorectal cancer more objectively, the meta-analysis of evidence-based medicine by systematically reviewing the existing literature is helpful to improve the reliability of traditional Chinese medicine decoction in the treatment of advanced colorectal cancer.

## 2 Methods and analysis

### 2.1 Study registration

The protocol has been registered on the INPLASY register. The registration number is INPLASY202080102 (Available from: https://www.doi.org). This protocol of meta-analysis will be reported in accordance with the preferred reporting items of the system review and meta analysis (PRISMA) statement guidelines.^[17,18]^

### 2.2 Data sources

We will independently search the following databases from their inception to July 2020: China National Knowledge Infrastructure (CNKI), China Biology Medicine (CBM), Wan Fang Data, the Chinese Science and Technology Periodical Database (VIP), PubMed, Cochrane Library, Embase, and Web of Science.

### 2.3 Eligibility criteria

#### 2.3.1 Types of study

We will include all randomized controlled trials of decoction combined with chemotherapy in the treatment of advanced colorectal cancer without language and blind restrictions.

#### 2.3.2 Type of participants

All included cases (over 18 years old) must be confirmed as advanced colorectal cancer by pathology or cytology. There will be no restriction on gender, race, or nation. Patients with non primary colorectal cancer or other tumors were excluded.

#### 2.3.3 Type of interventions

The RCTs that used traditional Chinese medicine decoction combined with chemotherapy will be included. There will be no restrictions on the types of traditional Chinese medicine decoction and chemotherapy.

#### 2.3.4 Type of comparators

In the same original study, the chemotherapy regimen in the control group of colorectal cancer patients is the same as that in the experimental group of colorectal cancer patients. The dosage and course of treatment are not limited.

### 2.4 Types of outcome measurements

The primary outcome will be an important basis for judging the curative effect after treatment. It will include:

1. DCR;
2. OS and FPS.

The secondary outcomes will include:

1. The quality of life (which is assessed according to the Kamofsky score);
2. Immune function assessment (that is to compare the levels of T cell subsets before and after treatment, including CD3 +, CD4 +, CD4 +/CD8 +, NK cells and other indicators Number average);
3. Evaluation of adverse drug reactions was evaluated according to NCI CTCAE version 4.0 (It mainly includes myelosuppression, gastrointestinal dysfunction, skin toxicity, liver, and kidney dysfunction and cardiac dysfunction).

### 2.5 Exclusion criteria

Any one of the following articles can be excluded:

1. The subjects had other primary tumors
2. Observational studies, animal experiments, case reports, reviews and other non randomized controlled trials or self-control study or random method error research investigate
3. Traditional Chinese medicine decoction combined with other treatments except chemotherapy
4. For the repetitive articles, only those with the latest publication year, large sample size and comprehensive information are retained
5. The full text cannot be obtained
6. Study on non oral administration of traditional Chinese medicine decoction.

### 2.6 Search strategy

The 2 authors will search the following databases (The retrieval time is from the establishment of each database to August 2020): PubMed, the Cochrane Library, Embase, Web of Science, China Biology Medicine (CBM), China National Knowledge infrastructure (CNKI), Wan Fang Data, the Chinese Science, and Technology Periodical Database (VIP). The group of search words was a combination of Colorectal Neoplasm (or Colorectal Tumor or Colorectal Carcinoma or Colorectal Cancer or Colonic Neoplasm or Rectal Neoplasm or Sigmoid Neoplasm or Anus Neoplasm) and traditional Chinese medicine. Take PubMed as an example to list the retrieval strategy, which will be attached as Annex 1 (https://kdocs.cn/l/soruAsZWisne).

### 2.7 Studies selection

**Figure 1:**
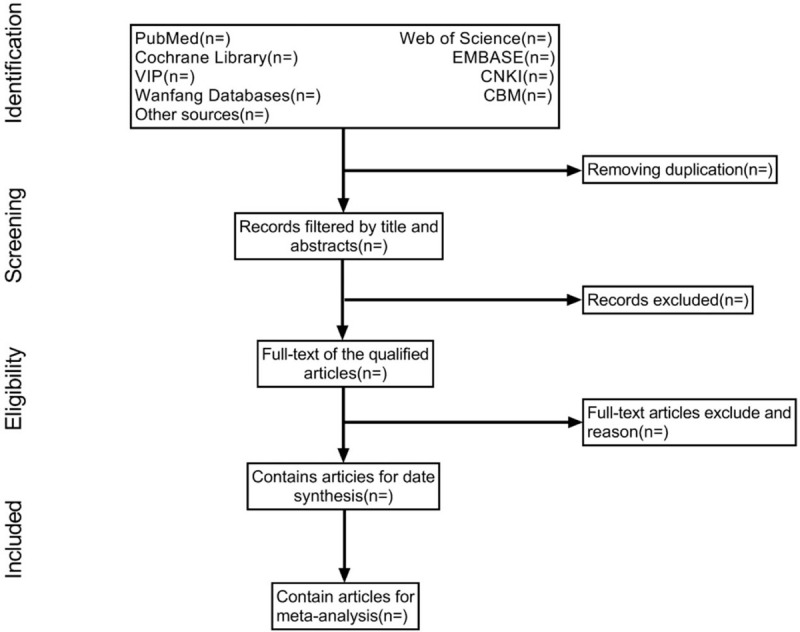
Flow diagram of studies identified.

We will use the software EndNote X9 to manage all retrieved studies. According to the pre-set criteria, the 2 researchers will independently exclude the obviously irrelevant literature, animal experiments, reviews, case reports, etc. After the first step of screening, the 2 researchers will obtain the full text for more detailed reading to determine whether they meet the inclusion criteria. After that, the 2 researchers cross checked the results of the included study. The divergent studies will be discussed by 2 researchers or decided by a third researcher. The procedures of study selection will be performed in accordance with the PRISMA flowchart (see Fig. 1).

### 2.8 Data extraction

Two reviewers will be responsible for information extraction according to the following information: The basic information of the included studies, including the first author, the year of publication, etc. The basic characteristics of the subjects, including the number of patients in the treatment group and the control group, gender composition, average age, intervention drug dosage, treatment course and other specific details. Outcome indicators and outcome measurement data needed for the study.

### 2.9 Assessment of risk of bias

Two researchers will select the quality assessment methods provided by Cochrane Handbook, including risk bias assessment form and Jadad modified scale. In case of disagreement, it will be decided by a third researcher. According to the characteristics of traditional Chinese medicine decoction and the methodology of randomized controlled trial, the following evaluation items will be adopted: random sequence generation, allocation concealment, blind methods, results data integrity, selective outcome reporting and other bias. As a result, the quality of evidence will be accepted as low risk, high risk, or ambiguous bias risk.

### 2.10 Data analysis

Relevant data will be performed by Revman 5.3 software provided by the Cochrane Collaboration and Stata 14.0 statistical software. Relative risk (RR) will be used for dichotomy results with 95% confidence intervals, and mean difference (MD) or normalized mean difference (SMD) will be used for continuous variables with 95% confidence intervals.

#### 2.10.1 Dealing with missing data

If the corresponding data cannot be obtained in the end, this kind of research will be eliminated. If the data cannot be obtained in the end, such research will be excluded.

### 2.11 Assessment of heterogeneity

The choice of random effect model or fixed effect model depends on the heterogeneity of the original research. In this study, the Cochrane Q test will be used to analyze the heterogeneity between studies, and *I*^2^ will be used to evaluate the heterogeneity. If there is no heterogeneity (*I*^2^ < 50%, *P* > .1), fixed effect model will be used in meta-analysis. Otherwise, we will choose sensitivity analysis, subgroup analysis or meta regression to explore the causes of heterogeneity. If the cause cannot be found and the degree of heterogeneity is acceptable, the random effect will be selected.

#### 2.11.1 Sensitivity analysis

Sensitivity analysis is to explore the impact of individual studies on aggregate results, which will be judged by the method of excluding studies one by one, so as to check the robustness of the comprehensive results.

#### 2.11.2 Additional analyses

In order to further study the heterogeneity, subgroup analysis and meta-analysis may be used to investigate the heterogeneity from age, gender, chemotherapy regimen, Chinese herbal decoction, sample sizes,

### 2.12 Publication bias

Funnel plot, Begg tests, Egger test will be used to investigate whether there is publication bias in this study. If the funnel plot is asymmetric, we will continue to process the asymmetric funnel plot through the shear compensation method, so as to ensure the symmetry of the funnel plot and eliminate publication bias.

### 2.13 Quality of evidence

Grading of Recommendations Assessment, Development, and Evaluation (GRADE) system will be used to evaluate the quality of evidence for the outcomes, Comprehensive results caused by 5 factors (risk of bias, inconsistency, indirectness, imprecision, publication bias) affect the quality of evidence, which is divided into 4 levels: high level, medium level, low level, very low level.^[19,20]^

### 2.14 Ethics and dissemination

No ethical approval is required, as SR will be based on published research. According to the PRISMA guidelines, SR's results will be published in a peer-reviewed scientific journal.

## 3 Discussion

As a disease endangering human health and life, colorectal cancer should be paid more attention. As a treasure of China, traditional Chinese medicine is also widely used in the treatment of tumors. However, there is still a lack of effective evidence that traditional Chinese medicine decoction is effective and safe in the treatment of colorectal cancer. Therefore, our study will evaluate the safety and effectiveness of TCM Decoction in the treatment of colorectal cancer through meta-analysis and Sr, so as to provide more reliable basis for clinical treatment of colorectal cancer.

## Acknowledgments

We would like to thank the members who have devoted themselves to this research.

## Author contributions

**Conceptualization:** Yan Yang.

**Methodology:** Xue-li Jia, Yu-xiang Sun.

**Supervision:** Si-miao Gong, Min Li.

**Writing – original draft:** Shu-fan Liao.

## Supplementary Material

Supplemental Digital Content
