# Supplementary material for: Efficacy and safety of traditional Chinese medicine decoction combined with chemotherapy in the treatment of advanced colorectal cancer: A protocol of a systematic review and meta-analysis
Source: Medicine (Baltimore). 2021 Jan 22;100(3):e23952. doi: 10.1097/MD.0000000000023952 (PMC7837827; doi:10.1097/MD.0000000000023952)
Supplement: Supplemental Digital Content [file medi-100-e23952-s001.doc]

Appendix 1

**Search strategy for Pubmed：**

#1 Colorectal Neoplasms[Mesh]

#2 Neoplasm, Colorectal[Title/Abstract] OR Colorectal Carcinoma[Title/Abstract] OR Carcinoma, Colorectal[Title/Abstract] OR Carcinomas, Colorectal[Title/Abstract] OR Colorectal Carcinomas[Title/Abstract] OR Colorectal Cancer[Title/Abstract] OR Cancer, Colorectal[Title/Abstract] OR Cancers, Colorectal[Title/Abstract] OR Colorectal Cancers[Title/Abstract] OR Colorectal Tumors[Title/Abstract] OR Colorectal Tumor[Title/Abstract] OR Tumor, Colorectal[Title/Abstract] OR Tumors, Colorectal[Title/Abstract] OR Neoplasms, Colorectal[Title/Abstract]

#3 #1 OR #2

#4 advanced

#5 Medicine, Chinese Traditional[Mesh]

#6 Traditional Chinese Medicine[Title/Abstract] OR Chung I Hsueh[Title/Abstract] OR Hsueh, Chung I[Title/Abstract] OR Traditional Medicine, Chinese[Title/Abstract] OR Zhong Yi Xue[Title/Abstract] OR Chinese Traditional Medicine[Title/Abstract] OR Chinese Medicine, Traditional[Title/Abstract] OR Traditional Tongue Diagnosis[Title/Abstract] OR Tongue Diagnoses, Traditional[Title/Abstract] OR Tongue Diagnosis, Traditional[Title/Abstract] OR Traditional Tongue Diagnoses[Title/Abstract] OR Traditional Tongue Assessment[Title/Abstract] OR Tongue Assessment, Traditional[Title/Abstract] OR Traditional Tongue Assessments[Title/Abstract]

#7 #5 OR #6

#8 randomized controlled trial[Publication Type]

#9 randomized controlled trial[Title/Abstract] OR random*[Title/Abstract] OR placebo[Tittle/Abstract]

#10 #8 OR #9

#11 #3 AND #4 AND #7 AND #10
